# Schizophrenia-like psychosis following left putamen infarct: a case report

**DOI:** 10.4076/1752-1947-3-7337

**Published:** 2009-07-14

**Authors:** Faisal Farid, Prem Mahadun

**Affiliations:** 1 Moorside Unit, Trafford General Hospital, Greater Manchester West Mental Health NHS Trust, Moorside Road, Urmston, Manchester, UK

## Abstract

**Introduction:**

Subcortical structures of the brain have been studied extensively to establish their implication in the development of psychotic symptoms in schizophrenia. Any pathology in these structures of the brain identified on neuroimaging techniques can give us helpful information in learning the neuropsychiatric background of psychotic symptoms in schizophrenia.

**Case presentation:**

We report an interesting case of a 38-year-old man with new onset psychosis who was found to have a lacunar infarct of the putamen region in the left basal ganglia on a computed tomography scan of his brain.

**Conclusion:**

It is possible to hypothesize that the psychotic symptoms in our patient may be the direct result of the putamen infarct, as pathology in the basal ganglia at the level of the striatum can result in complex cognitive and behavioural symptoms. Understanding organic causes of psychosis, including cerebrovascular compromises leading to damage of structures in the basal ganglia, can provide important information about the causality of psychosis and ways to treat it.

## Introduction

The basal ganglia and cerebellum have been extensively studied in schizophrenia as these areas of the brain are involved in the control of movement. Disease in these areas has been implicated in the pathophysiology of schizophrenia as movement disorders have been observed in patients with schizophrenia even in the absence of medications that induce movement disorders [1]. Basal ganglia diseases have been associated with a wide range of neuropsychiatric symptoms including depression, anxiety, delusions, apathy, irritability and disinhibition [2].

Neuroimaging studies conducted in the presence of focal cerebrovascular disorders, particularly involving the basal ganglia, can provide useful information about the neuropsychiatric background of schizophrenia symptoms. We report on an interesting case of schizophrenia-like psychosis in a patient with a putamen infarct.

## Case presentation

A 38-year-old Caucasian man was seen in the emergency department following a referral by his general practitioner, as there were concerns from his family that he was voicing bizarre ideas. He complained of feeling increasingly stressed, anxious and paranoid and he was having flashbacks of fictional events from his past. He described visualizing these events as if they were being played in a movie. He had beliefs that many people from his local community were involved in a conspiracy against him and he was being provoked by other people to get into fights. He also had beliefs that he was being followed and watched, and he elaborated that he could feel the presence of evil and evil things being passed onto him by other people.

He had no past psychiatric history and there was no family history of any mental health related problems. He had experimented with recreational drugs such as cocaine and ecstasy in the past but he denied any use of recreational drugs in the last 2 years. He worked as a successful self-employed businessman. His symptoms, particularly the persecutory delusions and delusional memory, worsened and he was admitted to the local psychiatric hospital.

His physical and neurological examination was unremarkable, and blood tests including urea and electrolytes, liver function tests and thyroid function tests were normal. An electroencephalogram (EEG) was inconclusive of any seizure activity and a computed tomography (CT) scan of his brain revealed a lacunar infarct of the putamen region in the left basal ganglia. A detailed history of any cardiovascular incidents was explored and he gave a history of severe crushing chest pain 6 months before presentation. He did not seek medical attention, the pain did not recur, and there were no cardiological or neurological sequels. An electrocardiogram and echocardiogram showed no compromise of cardiac functions.

The lacunar infarct was treated conservatively and he was commenced on an atypical anti-psychotic medication, aripiprazole, and his psychotic symptoms gradually improved over the following weeks. He tolerated aripiprazole quite well and did not experience any extrapyramidal or significant side effects to the medication. His insight into his delusional beliefs improved and he was able to rationalise them. Following 3 weeks of hospitalization, his symptoms had improved and he was successfully discharged with community follow-up.

## Discussion

Due to multifactor causality, the aetiology of psychotic illness remains a matter of much debate, and organic pathology including acute compromise of brain function has been investigated in many studies. Interesting observations have been noted when people with Parkinson's disease are treated with dopaminergic agents. They experience a variety of psychiatric symptoms such as hallucinations, delusions, mood elevation, nightmares and occasionally sexual behaviour disturbances. This marked overlap of movement disorders, neuropsychiatric symptoms and cognitive changes in patients with basal ganglia disorders is found to be related to disruption in the complex set of frontal subcortical circuits. These neuronal circuits link the cortex (predominantly frontal areas) to regions of the striatum, globus pallidus and/or substantia nigra and thalamus [3]. The striatum composed of putamen and caudate nucleus is the subcortical area that contains rich dopamine projections. Research thus far postulates that this neuromodulator contributes significantly to the pathophysiology of psychosis and its treatment with neuroleptic drugs through an impact on the corticostriato-thalamo-cortical loop [4].

Of the five basic frontal-subcortical circuits, the dorsolateral prefrontal circuit, orbitofrontal-subcortical circuit and anterior cingulate-subcortical circuit mediate important aspects of human behaviour [5]. These circuits mediate important aspects of cognition, emotion, civil behaviours and impulse control. Dysfunction of this region results in disinhibition, tactlessness, impulsivity and disrupted social interaction [6].

Our patient had an acute presentation with psychotic symptoms without any substance- induced, past or family psychiatric history. The lacunar infarct in the putamen area, a part of the striatum, on the CT brain scan indicates a possible role of organic cause in the onset of the acute psychosis.

## Conclusion

It is possible to hypothesize the psychotic symptoms in our patient may be the direct result of the putamen infarct, as pathology in the basal ganglia at the level of the striatum can result in complex cognitive and behaviour symptoms depending on which of the above mentioned brain circuits are affected. A previous case report highlights the emergence of psychotic symptoms in a patient with a right putamen infarct [7]. Interestingly, another case report identifies the resolution of psychotic symptoms in a patient with schizophrenia following haemorrhage in the left putamen [8].

Understanding of organic causes of psychosis including cerebrovascular compromises, infarction or ischaemia of basal ganglia can provide important information about the causality of psychosis and ways to treat it. Further research in this area will possibly lead to promising results.

## Consent

Written informed consent was obtained from the patient for publication of this case report. A copy of the written consent is available for review by the Editor-in-Chief of this journal.

## Competing interests

The authors declare that they have no competing interests.

## Authors' contributions

FF analysed and interpreted the patient data, neuroimaging findings and laboratory results, and also drafted the manuscript. PM was a major contributor in writing and revising the manuscript critically for important intellectual content. Both authors read and approved the final manuscript.
